# Expression of Inflammatory Mediators in Biofilm Samples and Clinical Association in Multiple Sclerosis Patients in Remission—A Pilot Study

**DOI:** 10.3390/life14030367

**Published:** 2024-03-11

**Authors:** Jakob Fehlhofer, Jutta Ries, Florian Tobias Nickel, Veit Rothhammer, Stefan Schwab, Marco Kesting, Mayte Buchbender

**Affiliations:** 1 Department of Oral and Maxillofacial Surgery, Friedrich-Alexander-Universität Erlangen-Nürnberg, 91054 Erlangen, Germany; jakob.fehlhofer@uk-erlangen.de (J.F.); jutta.ries@uk-erlangen.de (J.R.); marco.kesting@uk-erlangen.de (M.K.); 2 Department of Neurology, Friedrich-Alexander-Universität Erlangen-Nürnberg, 91054 Erlangen, Germany; florian.nickel@uk-erlangen.de (F.T.N.); veit.rothhammer@uk-erlangen.de (V.R.); stefan.schwab@uk-erlangen.de (S.S.)

**Keywords:** multiple sclerosis, cytokine expression, oral hygiene, periodontitis

## Abstract

Multiple sclerosis (MS) is a chronic inflammatory autoimmune disease of unknown etiology that affects the central nervous system and can lead to neurological impairment. Our aim was to determine whether MS patients also show inflammatory changes in the oral cavity more frequently than healthy individuals. For this purpose, we examined plaque samples for various mediators and their correlation with clinical findings. A study group (MS) and a control group were examined and compared. The plaque samples were analyzed for the expression of interleukins (IL-2, -6, -10), matrix metalloproteinases (MMP-7, MMP-9), and a surface antigen CD90 by quantitative real-time PCR. The clinical parameters examined were the Mombelli plaque index; bleeding on probing (BOP) index; periodontal pocket depth; and decayed, missing, and filled tooth (DMFT) index. The expression of MMP9 was significantly (*p* = 0.035) higher in the control group. The expression of IL-2 was increased four-fold in the MS group; however, this difference was not statistically significant. The mean PD (*p* < 0.001) and BOP index (*p* = 0.029) values were increased in the study group. The clinical parameters of the BOP index and PD were significantly amplified in the MS patients. However, no causal relationship between the investigated inflammatory mediators and the clinical findings could be established in this case series.

## 1. Introduction

Multiple sclerosis (MS) is a chronic inflammatory immune-mediated neurological disorder that is the most common reason (despite traumatic causes) for disability in young adults. The prevalence of MS in Europe is 127 per 100,000 inhabitants [1]. MS is nearly three times more common in females than in males. There is a higher prevalence in northern countries and the highest prevalence rates in both sexes for all countries occur between the ages of 35 and 64 [2]. The cause of multiple sclerosis remains mostly unknown. Genetic factors in combination with environmental factors (e.g., smoking, vitamin D level, EBV infection in adulthood) determine the disease risk [2,3,4,5]. MS is a progressive disease characterized by the inflammatory demyelination of axons in the central nervous system, which often (in 43–70% of patients) leads to cognitive impairment, such as memory, attention, and visual dysfunctions. Some of the physical impairments are fatigue, muscle weakness, muscle pain, and motoric dysfunction [6].

Periodontitis is a known inflammatory disease of the oral cavity. An imbalance of protective and pathogenic bacteria in the microbiome of the mouth can cause an overreaction of the immune system, which leads to an inflammatory loss of periodontal tissue. It is known that pro-inflammatory cytokines from pockets of inflammation in periodontitis can cause, in addition to local tissue loss, the promotion of systemic inflammation that may be correlated with other diseases (e.g., atherosclerosis, pregnancy complications, rheumatoid arthritis, inflammatory bowel disease) [7,8,9].

Some previous studies have shown that MS is associated with multiple oral health problems, as found in a systematic review from June 2019. Common problems mentioned were the impairment of the periodontal status and disorders in the temporomandibular area [10].

A study from Taiwan in 2013 provided evidence that there is a link between periodontitis and MS but only in female subjects. The results showed that MS patients were 1.86 times more likely to be diagnosed with chronic periodontitis [11]. 

Another study examination of the salivary profile and the DMFT index in MS patients showed significantly lower flow rates in these subjects and a nonstatistically significant higher DMFT index. Furthermore, the calcium and phosphorus levels differed between the study and the control group [12].

In 2015, dental and periodontal conditions in MS patients were examined depending on the expanded disability status scale (EDSS) score and the results showed that the plaque index, PD, and gingival index were higher in patients with a higher physical disability score [13]. The EDSS score is scaled from 0 to 10, with 0 indicating normal neurologic status without immobility, 9 indicating the highest form of immobility (i.e., being confined to bed), and 10 indicating death due to MS.

The examined indices are usually related to a higher risk of oral inflammation and periodontitis. 

Interleukins are known as important messengers for communication between leucocytes and the immune system. There are pro- as well as anti-inflammatory interleukins, both of which play a crucial role in the down- and upregulation of immune system activity and inflammatory processes. 

An examination of the mRNA levels of different interleukins in MS patients at the time of diagnosis and before the start of treatment showed reduced IL-2 and IL-10 expression. It has been suggested that the reduced levels of anti-inflammatory IL-10 play a role in MS and allow the proliferation of T-cells [14].

Multiple different cytokines and chemokines can be detected in the gingival crevicular fluid (GCF) and gingival tissue of patients with periodontitis. Proinflammatory cytokines, such as IL-6, are involved in the pathogenesis and progression of periodontitis. In the past, increased levels of proinflammatory cytokines, such as IL-2, IL-6, and anti-inflammatory IL-10, as well as tumor necrosis factor, were found in inflamed gingival sites [15,16]. 

IL-6 is known as an important proinflammatory cytokine with a major role in host defense. The previous literature has described that the modified expression of IL-6 interferes significantly with the pathogenesis of numerous inflammatory diseases and cancer [17]. 

Matrix metalloproteinases (MMPs) are enzymes involved in nearly all degradation processes of the human body, as well as the process of demyelination in MS. An upregulation of MMP-7 and MMP-9 was found in actively demyelinating MS lesions [18]. In the past, gingival biopsies of subjects with periodontitis showed positivity for the expression of MMP-7, -8, -9, and -13 [19].

Considering previous studies that have investigated a possible link between MS and poor oral health, different conclusions have been reached. Past findings showed that oral hygiene in patients with MS may be affected by impaired motor function and physical handicaps; however, there is no clear evidence that this also promotes inflammatory processes in the oral cavity more frequently in these patients. Since both multiple sclerosis and periodontitis are chronic inflammatory diseases, a possible systemic connection cannot be ruled out without further investigation. To determine whether patients with multiple sclerosis suffer more often from inflammatory changes in the oral cavity, we examined subjects suffering from multiple sclerosis with regard to their oral health. In addition, we examined the expression of various pro- and anti-inflammatory mediators in subgingival plaque samples and compared the results with those of healthy subjects.

## 2. Materials and Methods

### 2.1. Study Design and Patient Recruitment

In the present case series cohort study, a healthy control (HC) group and a cohort of patients in the study group with multiple sclerosis (MS) in remission were screened and compared. The subjects included in the study group had previously visited the Department of Neurology, University of Erlangen-Nuremberg, for the diagnosis and/or treatment of MS and the healthy subjects included in the control cohort were randomly selected from patients visiting for dental treatment in the Department of Oral and Maxillofacial Surgery, University of Erlangen-Nuremberg. Subjects were enrolled and examined from March 2019 to March 2023. Ethical approval (No.: 399_18 B) for this study was obtained from the ethics committee of the medical faculty of Friedrich-Alexander University Erlangen-Nuremberg, Germany, and the guidelines of the Declaration of Helsinki were followed. This study was also registered in the German clinical trial registry (DRKS00022956). Subjects and parameters from the control group were identical to those from previous publications by our department from our databases [8,9]. Adult patients of all ages and sexes provided informed consent before they were included in this study. The included subjects underwent dental examinations for approximately one hour each. During the examination, subgingival plaque samples were collected.

The exclusion criteria for both groups were as follows:

- All forms of nicotine abuse;
- Diabetes (type I and II);
- Previous diagnosis of periodontitis or treatment for periodontitis;
- Poor general health or disability that precluded detailed dental examination;
- Edentulousness.

### 2.2. Dental and Periodontal Parameter Examination and Biofilm Sample Collection

All subject plaque sampling and examinations and parameter recordings were carried out by one person (JF). Sampling was always performed according to a standardized protocol and procedure, as described in previous publications by our department and its affiliated laboratory [8,9].

The following clinical parameters were collected:

Parameter 1. Mombelli plaque index assessment:

grades from 0 to 3 (Grade 0: no plaque visible or detectable by probing; Grade 1: no plaque accumulation visible but detectable by probing the sulcus; Grade 2: visible plaque accumulation without probing; Grade 3: easily visible plaque accumulation even on the plain surfaces of the teeth);

Parameter 2. Bleeding on probing (BOP) index (in %) evaluation;

Parameter 3. Probing pocket depth measurement:

PD—the distance from the gingival margin to the bottom of the pocket—at 6 sites (mesiobuccal, buccal, distobuccal, mesiolingual, lingual, distolingual) for every tooth, except the 3rd molars;

Parameter 4. Decayed, missing, and filled tooth (DMFT) index procedure:

The two deepest periodontal pocket sites (>3 mm and <5 mm but not diseased in terms of periodontitis) in all four quadrants were selected for plaque sample collection. To avoid contamination with saliva, cotton swabs were used for isolation and two subgingival plaque samples were collected by inserting a sterile curette into the gingival pocket. The samples were then transferred to an Eppendorf tube, stored in a liquid nitrogen transport medium, and then placed in a freezer at −80 °C for a maximum of 15 min until further processing.

### 2.3. RNA Isolation

We isolated RNA from the subgingival plaque samples and determined the expression of inflammatory mediators (IL-2, IL-6, IL-10, MMP-7 and MMP-9, and CD-90) by using a reverse transcription quantitative polymerase chain reaction (RT-qPCR) with a positive control to ensure the presence of inflammatory material by integrin alpha L (CD11a). A common method for isolating RNA from biological samples is the TRIzol method. TRIzol, also known as TRI reagent, is a mixture of phenol and guanidine isothiocyanate. Added to biological samples, it lyses cells and denatures proteins that release RNA into the surrounding solution. RNA can be separated from other cellular components, e.g., DNA, lipids, and proteins, with further processing by adding chloroform and centrifugation. The aqueous phase can be removed by the addition of isopropanol. After the RNA is washed, dried, and dissolved in RNase-free water, it is ready for further procedures, such as reverse transcription, sequencing, or a PCR. Due to its simplicity and compatibility with a great variety of samples, this method is widely used.

To isolate RNA from our samples, we used this TRIzol™ method (Thermo Fischer Scientific, Waltham, MA, USA). The transcription of RNA into cDNA was performed using a quantitect reverse transcriptase kit (Qiagen, Venlo, The Netherlands).

### 2.4. RT-qPCR

With the cDNA available, the expression of the mediators of interest could be determined by performing a qPCR of our genes of interest (IL-2, IL-6, IL-10, MMP-7, MMP9, and CD90, as well as a housekeeping gene, GAPDH) by using primer sets from Qiagen (Venlo, The Netherlands; Table 1), according to manufacturer’s instruction. For each PCR run, a spleen sample was included as a positive control.

### 2.5. Correlation of Selected Inflammatory Markers with Dental and Periodontal Variables

We calculated the average cycle threshold (CT) for each sample. All samples were analyzed as duplicates in the qPCR analysis. For further analyses, we used only samples with a CT value <37 and clear amplification of the housekeeping gene GAPDH. Samples that showed a higher CT value for the endogenous control were not considered in further analyses as it must be assumed that the quality of the obtained sample was insufficient. The samples that showed an insufficient CT value for the target gene but an acceptable value for GAPDH were classified as undetected and set to a CT value of 50 that corresponds to the maximum number of PCRs [20,21].

Using GAPDH as an endogenous control, we normalized the CT values of the samples. These data (∆CT values) were used for the subsequent statistical analysis. 

The ∆CT values provide information on the expression of the investigated genes. A high ∆CT value indicates low expression of the gene (↑∆CT = ↓expression). Fold change was calculated by the ∆∆CT method to determine the relative change in the expression between the two groups. (Formula 2^−ΔΔCT^) [21].

To minimize the possible bias of sampling from the pocket, only one practitioner (JF) took the samples. The study size was not statistically determined in advance because of the pilot study character. The primary outcome of this study was defined as a difference in the inflammatory markers in terms of their expression levels in the pockets of each group. Secondary outcomes were the assessment of clinical parameters (MPI, BOP index, PD, DMFT index) and their correlation with inflammatory markers.

### 2.6. Statistical Analysis

The collected data were statistically analyzed using SPSS, Version 26 software (IBM, Armonk, New York, NY, USA). Unless otherwise stated, the results are presented as the mean values (±standard deviation). For statistical comparisons between groups, the nonparametric Kruskal–Wallis test was performed. Differences between groups were considered significant if the *p*-value was <0.05. If a statistically significant difference was found, then, we performed an additional pairwise comparison with a separate nonparametric Mann–Whitney U test. For statistical comparison of the data (clinical parameters and expression) between groups, a nonparametric Mann–Whitney U test with a significance level of *p* < 0.05 was performed. Post hoc analysis was performed to evaluate the test power. 

## 3. Results

### 3.1. Study Participants’ Characteristics

In total, twenty subjects divided into two equally sized groups (HC group *n* = 10, MS group *n* = 10) were included in this study. The HC group consisted of *n* = 6 female and *n* = 4 male patients with a mean age of 46 ± 12 years and the MS group consisted of *n* = 6 female and *n* = 4 male patients with a mean age of 39 ± 9 years. The two groups were matched in terms of sex but not age. The time of the first diagnosis of MS varied between 2001 and 2022, as seen in Table 2. All the patients in the MS group received medication for the treatment (mitoxantrone *n* = 1; peginterferon beta-1a *n* = 1; ocrelizumab *n* = 4; natalizumab *n* = 2; glatiramer acetate *n* = 1, dimethyl fumarate *n* = 1) illustrated Table 2. Of the patients suffering from MS, *n* = 7 were diagnosed with the relapsing-remitting type, *n* = 2 were diagnosed with the secondary progressive type, and *n* = 1 was diagnosed with the primary progressive type (Table 2). None of the subjects from each group presented visible changes in the oral mucosa. The patient with the highest EDSS score of 6.0 (*n* = 1) did not report any physical limitations in his daily oral hygiene due to the underlying disease. The other patients (*n* = 9) had an EDSS score < 4.0 (Table 2).

### 3.2. Dental and Periodontal Analysis

#### 3.2.1. Mombelli Plaque Index

The mean value for the MPI was not significantly different between groups. With a mean value of 1.17 ± 0.79 for the HC group and 1.53 ± 0.84 for patients from the MS group (Table 3).

#### 3.2.2. Bleeding on Probing (BOP) Index

The BOP index was significantly higher (*p* = 0.029) in the MS group than in the group of healthy subjects. Twenty-two percent of all investigated sites in the MS group were positive for bleeding on probing whereas 11.92% of the sites in the HC group were positive (Table 3).

#### 3.2.3. Pocket Depth

The mean PD in the HC group was 2.44 ± 0.77 mm, which was significantly (*p* ≤ 0.001) less than the measured PD of 2.70 ± 0.82 mm in the MS group (Table 3).

#### 3.2.4. DMFT Index

The mean values for the DMFT index differed between groups, although the difference was not statistically significant (*p* = 0.571). The mean DMFT index was 13.8 in the HC group and 15.5 in the MS group (Table 3).

### 3.3. Analysis of Selected Gene Expression

The mean ΔCT values for all investigated mediators are presented in Table 4.

#### 3.3.1. Pro- and Anti-Inflammatory Cytokines

With a fold change of four, the expression of the proinflammatory IL-2 was higher in the MS group than in the HC group. The difference in the expression of IL-2 was not statistically significant (*p* = 0.386). IL-10, as an anti-inflammatory mediator, showed lower expression (ΔCT IL-10 = 20.64 ± 5.20) in the MS group. 

#### 3.3.2. Matrix-Metalloproteases

A statistically significant difference (*p* = 0.035) in the expression of the marker MMP-9 was identified by its ΔCT values, with a lower value for the HC group. Additionally, MMP-7 showed lower values in the HC group (ΔCT MMP-7 = 15.14 ± 8.74). 

#### 3.3.3. CD11a and CD90 Proteins

CD11a, which served as a positive control for inflammatory material presence, was lower in the HC group. The ΔCT values for CD90 were 16.66 ± 6.77 in the HC group and 20.09 ± 4.96 in the MS group (*p* = 0.383). 

## 4. Discussion

General diseases with an inflammatory pathogenesis, involving inflammatory markers, are playing increasingly important roles for dentists. Changes in the oral microfilm occur due to the disease’s underlying mechanisms and to restrictions resulting from the disease itself, leading to periodontium changes, which can also lead to tooth loss [8]. Therefore, there may also be a connection to the oral biofilm in the case of MS.

In this case series, we showed that the clinical parameters (MPI, BOP index, PD, and DMFT index) were on average higher in the MS group than in the healthy control group. The difference regarding the BOP index and PD was significant and, with the inclusion of the MPI and DMFT index, a clear tendency for elevated clinical inflammatory parameters was observed in MS patients. Other studies also reported higher DMFT index values, numbers of dental caries, and temporomandibular disorders in patients suffering from MS [12,22,23]. Even though the mean PD measured in our study was deeper in the study group than in the control group, this value should not be considered pathological and is not the sole indicator of the presence of periodontitis.

These results are also comparable with a study of MS patients regarding oral health, whereby the subdivision was made based on the current EDSS score. Subjects with a higher EDSS score showed higher values for the PI, PD, and GI indices [13]. This is explained by the impaired mobility of such patients with an influence on mouth cleaning as well. Since only one patient included in our study population had an EDSS score > 5, we can exclude this as an influencing factor on clinical parameters. Thus, one might think that it is not the physical limitation per se but an inflammatory reaction that causes the increase in the BOP index and PD.

In the past, IL-6 has been linked to the acute and initial phases of periodontitis with significant overexpression. Further progression of periodontitis did not interfere with the expression of IL-6 [24]. As we observed a significantly higher BOP index as a sign of an acute inflammation process, we expected the expression of IL-6 to be higher in the MS group. However, the fact that we found a nearly similar expression of IL-6 in both groups (a high ∆CT value indicates low expression of the gene (↑∆CT = ↓expression) may be linked to the usage of natalizumab as a therapeutic for MS. It has been observed that natalizumab can markedly decrease proinflammatory cytokines, such as IL-1beta, IL-6, and IL-8, in the cerebrospinal fluid and even in the circulating plasma [25]. Nevertheless, even after a new analysis without the affected patients, the results remained without significance.

An investigation into circulating interleukins and their association with MS showed evidence that elevated levels of IL-2Rα confer an increased risk of developing MS [26]. With regard to periodontitis, there has long been evidence that serum IL-2 may be an indicator of disease activity in periodontitis as higher levels of IL-2 are observed in these patients [27]. Thus, elevated IL-2 could be seen in both diseases and was an important parameter for our study aim. The MS group in our study showed a fold change of four regarding the measured IL-2 in the subgingival plaque samples. However, the differences in the expression of IL-2 were not significant but higher in the gingival pocket of MS patients. Considering this result and the knowledge from previous investigations concerning IL-2, there could be a possible connection between the diseases. 

IL-10 is known as an anti-inflammatory cytokine of the human body that can effectively downregulate inflammatory processes and the production of proinflammatory cytokines, such as IL1, IL-6, or TNF-α [28,29]. A co-influencing factor for decreased expression of proinflammatory markers is smoking, which was confirmed in a comparison of smokers to nonsmokers, with significantly reduced TNF levels found in smokers. However, to eliminate such an effect in both groups, smokers were excluded [30]. In MS patients in a remission phase, it was found that higher levels of IL-10 can occur. A study from 2019 showed that the level of IL-10 in healthy subjects and in MS patients in the remission phase was higher than that in subjects with progressive MS [31]. Since all patients from the MS group were in remission and none of the subjects from the HC group had periodontitis, this may explain the similar expression of IL-10 in the two groups. Furthermore, the bone-protective effect of IL-10 and the increase in IL-10 in the remission phase in MS could explain why relatively low PD with otherwise elevated clinical parameters of the MPI and BOP index (which can be seen in patients with signs of gingivitis) were present in our study [32]. In addition, since IL-10 is known to inhibit the production of the proinflammatory cytokine IL-6 induced by *Porphyromonas gingivalis*, the approximately equal expression of IL-10 in both groups could also be a reason for the similar expression of IL-6 in the pocket samples [33].

Surprisingly, in this study, the expression levels of MMP-7 and MMP-9 were lower in the MS group (=higher ∆CT values). We assumed there would be higher expression of MMPs in our MS samples as the literature shows that MMPs play a crucial role in the progression and process of demyelination and are often more highly expressed in these patients than in healthy people [18,34,35,36,37]. The fact that there are several immunomodulatory drugs used in the treatment of MS may explain the lower expression of MMP-7 and MMP-9 in our study group. In 2020, the effect of natalizumab use on MMPs in a mouse model was studied and the results showed that natalizumab significantly reduced the expression of MMPs and that their tissue inhibitors (TIMPs) were upregulated [38]. Another study on natalizumab showed that this drug significantly decreases the levels of MMP-9, MMP-2, MMP-3, MMP-8, and MMP-10 when used in humans [39]. As most of the therapeutics used to treat MS target the peripheral immune response, a current approach is to target disease-promoting processes, such as the reduction of MMPs. Fingolimod has been successfully used to reduce the frequency of relapses and the activity of MS. In addition, this drug was found to be effective in regulating MMP-9; however, in our study population, only one patient received this drug in the past. The drugs currently used for therapy in MS are immunomodulatory and immunosuppressive drugs, monoclonal antibodies, or antimetabolites and mostly inhibit B and T lymphocytes, as well as monocytes and macrophages. Well-known representatives of the drugs are glatiramer acetate, mitoxantrone, fingolimod, natalizumab, and ocrelizumab [40]. All these common drugs for the treatment of MS were used in our study group. The medication of the subjects in the study group could explain the expression of MMPs; however, to be able to make reliable statements about this, it must be evaluated in larger sample-sized studies. A study with an additional cohort of recently diagnosed, treatment-naïve MS patients would be interesting and useful.

Some more limitations other than the sample size should be mentioned and discussed. First of all, the group of MS patients is not homogeneous enough to derive reliable statements due to the different subtypes. Moreover, medication, such as biologicals, might have had a larger confounding effect on cytokine expression and the bacterial composition in these cases, which was not analyzed. However, this process could have a decisive influence on the release of cytokines and, thus, on the course of MS. In addition, there might be a bias owing to the preselection of markers, especially the clinical parameters, as they do not provide information about the severity of the damage to the periodontium. In addition, the severity of MS, i.e., the form, will also be able to show an influence on the microbiome, which was also inconclusively assessed here. Moreover, the lack of matching the patients in terms of age limits the validity of the results. However, with this case series, a connection between these two diseases seems more than possible.

The study of the microbiome in patients with chronic inflammatory diseases is an important consideration and should be included in future studies.

## 5. Conclusions

The clinical parameters of the BOP index and PD in MS patients were significantly higher than those in healthy controls whereas the expression of MMP-9 was significantly decreased. Further studies with larger sample sizes and an analysis of the oral microbiome would provide further important insights into the connection to MS.

## Figures and Tables

**Table 1:** Details and product information of the used quantitect primer assays [9].

Marker	Product Name	Cat. No.	Lot No.
IL-2	Hs_IL2_1_SG	QT00015435	354840918
IL-6	Hs_IL6_1_SG	QT00083720	315797203
IL-10	Hs_IL10_1_SG	QT00041685	315797224
MMP7	Hs_MMP7_1_SG	QT00001456	315797201
MMP9	Hs_MMP9_1_SG	QT00040040	354840919
CD90	Hs_THY1_1_SG	QT00023569	354840947
CD11a	Hs_ITGAL_1_SG	QT00034006	361111662
GAPDH	Hs_GAPDH_1_SG	QT00079247	345551798

**Table 2:** Presenting the detailed information for the MS group about sex, age, diagnosis, form of MS, EDSS score, and therapy.

No.	Sex	Age	Initial Diagnosis	Expanded Disability Status Scale (EDSS)	Type of Multiple Sclerosis	Therapy
1	W	49	2012	0	Relapsing-Remitting MS	Interferon beta-1a
2	W	46	July 2015	5	Secondary Progressive MS	Mitoxantrone
3	M	47	October 2012	4	Relapsing-Remitting MS	Ocrelizumab
4	W	53	2018	3.5	Primary Progressive MS	Ocrelizumab
5	M	27	July 2021	2	Relapsing-Remitting MS	Natalizumab
6	M	59	2012	4.5	Secondary Progressive MS	Natalizumab
7	W	37	2001	4	Relapsing-Remitting MS	Ocrelizumab
8	M	41	March 2015	4	Clinically isolated Syndrome	Ocrelizumab
9	M	39	July 2021	0	Relapsing-Remitting MS	Glitaramer acetate
10	W	49	April 2020	1	Relapsing-Remitting MS	Dimethyl fumarate

**Table 3:** Presenting the mean values as well as the standard deviations for the clinical parameters MPI, BOP, PD, and DMFT for the HC group and the MS group. The corresponding *p*-values and test power values are presented as well. The parameters in bold were shown to be significant in the statistical analysis.

	HC Group *n* = 10	MS Group *n* = 10	*p*-Value	Test-Power
Mombelli PI (grade 0–3)	1.17 ± 0.79 (*n* = 10)	1.53 ± 0.84 (*n* = 10)	0.178	-
**BOP (in %)**	11.92 ± 7.56 (*n* = 10)	22.00 ± 10.92 (*n* = 10)	**0.029**	**0.769**
**PD (mm)**	2.44 ± 0.77	2.70 ± 0.82	**<0.001**	**-**
DMFT	13.80 ± 7.94	15.50 ± 4.81	0.571	0.115

**Table 4:** Presenting the mean values, as well as the standard deviation, for the ΔCT values of the investigated mediators (IL-2, IL-6, IL-10, MMP-7, MMP-9, CD90, and CD11a) for the HC group and the MS group. The corresponding *p*-values, test power values, and fold change values are presented as well. The parameters in bold were shown to be significant in the statistical analysis. A high ∆CT value indicates low expression of the gene (↑∆CT = ↓expression).

	HC Group *n* = 10	MS Group *n* = 10	*p*-Value	Test-Power	Fold Change
∆CT IL-2	21.63 ± 2.79 (*n* = 10)	19.51 ± 6.95 (*n* = 10)	0.386	0.157	4
∆CT IL-6	16.77 ± 6.53 (*n* = 10)	18.65 ± 5.51 (*n* = 10)	0.497	0.120	4
∆CT IL-10	19.79 ± 5.16 (*n* = 9)	20.64 ± 5.20 (*n* = 10)	0.726	0.070	1
∆CT MMP7	15.14 ± 8.74 (*n* = 9)	18.74 ± 7.50 (*n* = 10)	0.353	0.130	12
**∆CT MMP9**	7.54 ± 3.72 (*n* = 9)	14.56 ± 8.56 (*n* = 10)	**0.035**	**0.662**	**130**
∆CT CD90	16.66 ± 6.77 (*n* = 10)	20.09 ± 4.96 (*n* = 10)	0.214	0.383	11
∆CT CD11a	10.92 ± 7.87 (*n* = 9)	15.11 ± 6.99 (*n* = 10)	0.240	0.368	18

## Data Availability

The datasets used and/or analyzed during the current study are available from the corresponding author upon reasonable request.
