# Correction: Fehlhofer et al. Expression of Inflammatory Mediators in Biofilm Samples and Clinical Association in Multiple Sclerosis Patients in Remission—A Pilot Study. *Life* 2024, *14*, 367

**DOI:** 10.3390/life14080932

**Published:** 2024-07-25

**Authors:** Jakob Fehlhofer, Jutta Ries, Florian Tobias Nickel, Veit Rothhammer, Stefan Schwab, Marco Kesting, Mayte Buchbender

**Affiliations:** 1 Department of Oral and Maxillofacial Surgery, Friedrich-Alexander-Universität Erlangen-Nürnberg, 91054 Erlangen, Germany; 2 Department of Neurology, Friedrich-Alexander-Universität Erlangen-Nürnberg, 91054 Erlangen, Germany

**References**

The reference 22 was deleted in the original publication [1]. With this correction, the order of some references has been adjusted accordingly. The authors state that the scientific conclusions are unaffected. This correction was approved by the Academic Editor. The original publication has also been updated.
